# READ TO SUCCEED: HOW BOOK CLUBS CAN IMPROVE INTERGENERATIONAL RELATIONS AND FOSTER AGE FRIENDLINESS IN COMMUNITIES

**DOI:** 10.1093/geroni/igad104.2641

**Published:** 2023-12-21

**Authors:** Katelyn Frey, Jennifer Sublett, Nikolina Kravljaca, Jennifer Stanley

**Affiliations:** University of Akron, Akron, Ohio, United States; University of Akron, Akron, Ohio, United States; The University of Akron, Barberton, Ohio, United States; University of Akron, Akron, Ohio, United States

## Abstract

Intergenerational programming is recommended to improve age friendliness in communities, but there is little empirical research to support its effectiveness (Canedo-García et al., 2017). This project examined whether a multimodal intergenerational book club challenged age stereotypes and reduced ageism among participants (N=41). Twenty-one older adults (61-82) and 20 younger adults (18-22) read A Man Called Ove and met three times to introduce themselves (Time 1) and discuss the book (Time 2 and 3). Before the first meeting and after subsequent meetings, participants completed a survey to assess ageist beliefs and stereotypes. We expected ageist beliefs and stereotypes to decrease across time during the book club program. There was a statistically significant interaction between age group and time on benevolent ageism F(2, 52)= 5.23, p= .009 , partial η2= .167, such that benevolent ageism was more endorsed in younger adults (M=6.64, SE= 2.48, p= .011) compared to older adults at Time 1. This difference was not observed at Time 2 or Time 3 due to younger adults’ decreased overall endorsement. For positive generational stereotypes about older adults, there was a statistically significant main effect of time, F(2, 52)=7.095, p=.002, partial η2= .214, such that both younger and older adults increased their endorsement of these stereotypes from Time 1 to Time 3; similar findings occurred for positive stereotypes about younger adults F(2, 46)=4.53, p=.016, partial η2= .165. Results indicate intergenerational book clubs are an effective means to improve intergenerational relations and reduce ageist attitudes in younger adults.

